# Editorial: Physiological, biochemical and molecular approaches in response to abiotic stresses in plants

**DOI:** 10.3389/fpls.2023.1194937

**Published:** 2023-04-21

**Authors:** Giselle Camargo Mendes, Caroline Müller, Andréa Miyasaka Almeida

**Affiliations:** ^1^ Departamento de Biotecnologia, Instituto Federal de Santa Catarina, Lages, Brazil; ^2^ Programa de Pós-Graduação em Ciência e Tecnologia Ambiental, Universidade Federal da Fronteira Sul, Erechim, Brazil; ^3^ Centro de Genómica y Bioinformática, Universidad Mayor, Santiago, Chile

**Keywords:** drought, high temperature, salinity, transcriptome, metabolome, signaling response, ubiquitination, plant growth promoting bacteria

As sessile organisms, plants are constantly exposed to abiotic stresses such as drought, extreme temperatures, and high salinity which negatively affect their growth and development. The response to abiotic stress is multigenic with complex signaling pathways involving hormones, specific transcription factors, and second messengers such as Ca^2+^. There are signaling pathways that are common to different abiotic stresses and plant species, and those that occur only in response to specific stresses. Morphological and physiological responses are triggered at the end of these signaling pathways. Endemic and cultivated plants differ in their response to stress. There are several endemic species that are extremely tolerant to various abiotic stresses and exhibit characteristics that could be transferred to cultivated plants. Knowledge of the molecular and physiological responses to various abiotic stresses could provide the basis for potential applications to improve tolerance through genetic engineering, marker-assisted breeding, grafting and other sustainable approaches. Many responses to the abiotic stressors discussed in this topic are summarized in **Figure 1**.

**Figure 1:**
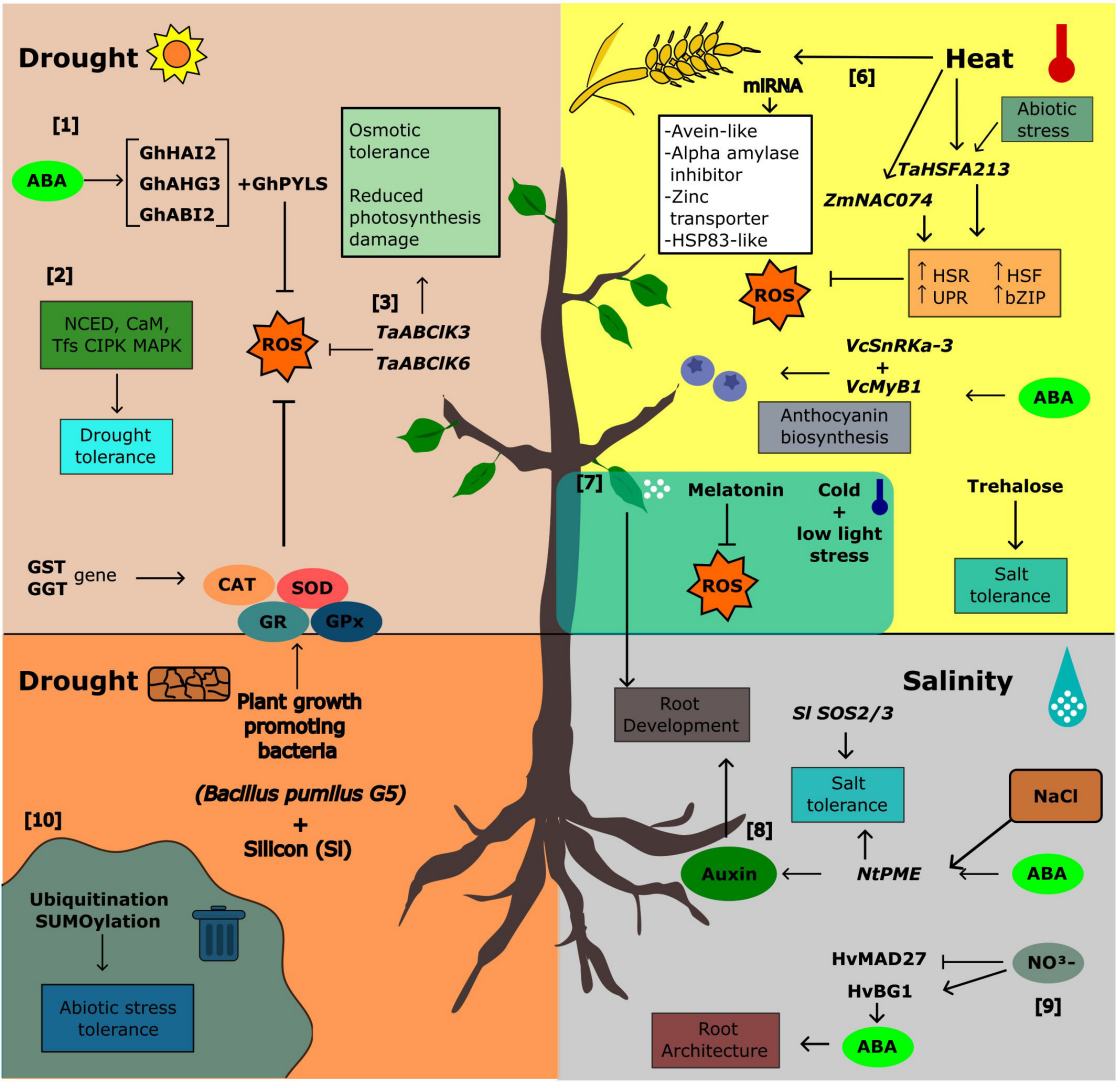
Integration of signals in abiotic stress and adaptive responses in plants. [1] The sensitivity and synthesis of ABA were regulated by the GhHAI2, GhAHG3, and GhABI2 genes in cotton (Shazadee et al.), and [2] NINE-CIS-EPOXYCAROTENOID DIOXYGENASE (NCED) and zeaxanthin epoxidase (ZEP) in two xerophytic desert trees (Yang and Lv). In these trees, signaling pathways under drought stress also involves CaM, CIPK, and MAPKK genes (Yang and Lv), and [3] overexpression of TaABC1K3 and TaABC1K6 in Arabidopsis and wheat improved drought tolerance (Gao et al.) by inducing osmotic tolerance and reduced photosynthetic damage. In addition, drought stress tolerance is also promoted by [4] application of plant growth-promoting bacteria (PGPB) and silicon (Si) which increase antioxidant enzymes such as catalase (CAT), superoxide dismutase (SOD), GR, and GPx and regulate GST and GGT genes in Glycyrrhiza uralensis (Ma et al.). [5] Low light and low-temperature stress could be mitigated by melatonin foliar application in pepper by controlling root development and ROS production (Li et al.). [6] In heat stress, ZmNAC074 from maize (Xi et al.) and TaHsfA2-13 from wheat (Meng et al.) were important genes related to heat shock response (HSR), unfolded protein response (UPR), basic leucine zipper (bZIP), heat shock proteins (HSP), and reactive oxygen species removal, conferring tolerance to these plants. Heat stress tolerance has also been observed through the upregulation of avenin-like cysteine-rich storage proteins, amylase inhibitors, zinc transporters, and HSP83-like proteins expressed by miRNA in the early stages of wheat seed development (Paul et al.). [7] In blueberry fruit, ABA is also involved in anthocyanin biosynthesis (VcMYB1 and VcSnRK2.3) (Wang et al.). [8] Modulation of root system architecture is also mediated by abscisic acid (ABA) in tomato (Zinta et al.) and barley (HvMADS27 x ABA) (Smoczynska et al.). NtPME genes in tobacco roots were also induced by ABA and auxin pathways, mediating root development under salinity (Sun et al.). [9] Release of ABA from conjugates can also inhibit root development under conditions of nitrogen excess in barley (Smoczynska et al.). [10] Other post-transcriptional processes such as ubiquitination and SUMOylation play an important role in tolerance to various abiotic stresses (Singh et al.).

Drought, exacerbated by climate change in recent decades, is one of the major stresses affecting the growth and productivity of various plant species. Abscisic acid (ABA) plays a central role in the response of plants to drought and other abiotic stresses. Analysis of full-length transcripts from two xerophytic desert trees showed that three genes for zeaxanthin epoxidase (ZEP) and one gene for NINE-CIS-EPOXYCAROTENOID DIOXYGENASE (NCED), gene, a rate-limiting enzyme in the ABA biosynthetic pathway, were up-regulated, which could explain the increase in synthesis of ABA during stress (Yang and Lv). In addition, the authors reported that signaling pathways involving *CaM*, *CIPK*, and *MAPKK* genes were upregulated in these species. In a later work, Yang and Lv observed 217 differentially expressed genes (DEGs) belonging to the WRKY, Maf, ER, MYB, and FAR1 transcription factor (TF) families in these xerophytic desert plants under drought stress. TFs are important for regulating gene expression networks of target genes and the expression of different families reveals their central role in the complex regulatory network of drought tolerance.

In cultivated cotton, members of the ABA pathway, such as 2C-type protein phosphatases (PP2Cs), were shown to act as negative regulators in the osmotic stress response through ABA-mediated signaling (Shazadee et al.). According to the authors, *GhHAI2*, *GhAHG3*, and *GhABI2* showed altered phenotypes in response to osmotic stress (induced by polyethylene glycol) through changes in leaf physiology and root morphology and regulation of reactive oxygen species (ROS) degradation in the genetic VIGS (virus-induced gene silencing) studies.

Silencing of *PP2C* genes in this species significantly increased the expression of ABA-dependent stress marker genes and resulted in altered phenotypes in response to osmotic stress (Shazadee et al.). Twenty-nine and 41 *PP2C* genes were also detected in *Haloxylon ammodendron* and *Haloxylon persicum*, two species of xerophytic trees, in addition to one *PYL9* and one *PYL8* and one and four *SnRK2* genes, that were upregulated, indicating their role in the ABA-signaling regulatory system, which plays an important role in the drought tolerance process (Yang and Lv).

Gao et al. performed a comprehensive genome-wide analysis of the BC1 complex kinase (ABC1K) activity gene family of wheat using the newly released published wheat genome database (IWGSC RefSeq v2.1). The genome-wide analysis identified 44 wheat ABC1K family genes that contained the typical ABC1K kinase domain and three (I–III) clades. Overexpression of *TaABC1K3* and *TaABC1K6* in yeast and *Arabidopsis* significantly improved drought tolerance. Moreover, *TaABC1K3* and *TaABC1K6* were shown to play essential roles in scavenging ROS and mitigating photosynthetic damage caused by drought stress. In addition, wheat ABC1K genes such as *TaABC1K3 and TaABC1K6* have numerous cis-elements associated with environmental stress, including ABRE, MBS, and G-box, which may play important roles in defending against various abiotic stresses.

Despite the activation of various cellular signaling pathways for plant resistance to drought stress, this may lead to excessive accumulation of ROS as secondary stress that causes oxidative damage in plants. Under stress conditions, plants tend to increase the production of antioxidant enzymes and non-enzymatic components such as ROS and the AsA-GSH cycle, which may contribute to plant tolerance to drought. Assessment of the antioxidant system was important to determine the role of synergistic application of plant growth-promoting bacteria (PGPB) and silicon (Si) in alleviating drought stress in *Glycyrrhiza uralensis*, a perennial herb widely distributed in arid and semiarid desert steppes and commonly used in Chinese folk medicine (Ma et al.). Using physiological, transcriptomic, and metabolomic techniques, the authors demonstrated that PGPB in combination with Si significantly increased the activities of SOD and CAT. The combination of *Bacillus pumilus* (G5) and Si further enhanced GSH formation by increasing the activities of GR and GPX and upregulating the *GST* and *GGT* genes, which contributed to a reduction in oxidative stress (Ma et al.).

Like drought stress, tolerance to heat stress (HS) is a highly complex process involving multiple cell signaling pathways. There are several types of TFs that act as master regulators and coordinate the expression of genes involved in the response to heat stress, such as the NAC proteins (NAM, ATAF1/2, and CUC2). In particular, the transcription factor *ZmNAC074* isolated from maize was upregulated after 8-h exposure to heat stress (42°C) (Xi et al.). The authors also confirmed the increase in tolerance to heat stress by overexpression of *ZmNAC074* in *A. thaliana* and discovered genes related to heat shock response (HSR), unfolded protein response (UPR), and removal of ROS, as well as the interaction proteins between different transcription factors such as bZIP (basic leucine zipper) and HSF (heat shock factor) TFs in response to heat treatment. Also using wheat plants, Meng et al. analyzed the heat shock transcription factor *TaHsfA2-13* cloned from wheat and found that its expression, as well as overexpression of *TaHsfA2-13* in the Col-o or *athsfa2* mutant of *A. thaliana* was up-regulated by heat stress, H_2_O_2_, mannitol, and salinity, suggesting that this gene may increase tolerance to various abiotic stresses in wheat plants.

Paul et al. used the transcriptome to analyze wheat plants exposed to thermal stress during the grain filling stage and identified 309 DEGs from 115,656 conserved genes involved in processes such as signal transduction, starch synthesis, the antioxidant system, and conserved and uncharacterized putative genes that respond to heat stress. Among the major proteins, the authors detected upregulation of avenin-like cysteine-rich storage proteins expressed in the early stages of seed development; amylase inhibitors responsible for protecting seed starch reserves from degradation into simpler oligosaccharides; aspartic proteinase, which plays an important role in protein processing in response to various stresses; elongation factor 1-alpha, a multifunctional protein that transcribes elongation factors, which play an essential role in mediating critical cellular processes related to cell growth, proliferation, and differentiation by interacting with other cellular proteins; and α-gliadin, heat shock protein, and zinc transporter, which have been described to confer heat stress tolerance in plants.

Salinity has steadily increased in irrigated areas, significantly affecting crop yields. Guo et al. conducted a comprehensive review on the morphological, physiological, and biochemical effects of salt and discussed in detail the mechanisms of salt tolerance in tomato, the most widely grown fruit crop in the world. In particular, the exogenous application of trehalose (Ter), a non-reducing sugar known to be an osmoprotective molecule, was able to reverse the negative effects on photosynthetic rate and Calvin cycle enzymes in tomato plants (Yang et al.). In addition, the authors reported that Ter increased transport selectivity and ion levels and affected the expression of genes related to ion homeostasis under salt stress conditions. Tobacco plants overexpressing the gene *NtPME043*, which is related to the enzyme pectin methylesterase (PME), an important cell wall component, showed greater root growth after three weeks of salt treatment, indicating greater tolerance to this abiotic stress (Sun et al.).

In countries with subtropical climates and very cold winters, the cultivation of vegetables such as peppers, can suffer from the effects of low light and low temperatures, even in protected facilities (Li et al.). These authors showed that the application of melatonin, an important hormone that acts as a secondary metabolite, mitigate low light and low temperature stresses by modulating of root growth and improving antioxidant defense systems in this species.

Abiotic stress also affects the availability and uptake of nutrients by plants. Nitrogen (N) is the macroelement that plants require in greater amounts. In a comprehensive review of root system architecture for tolerance to various abiotic stresses in potato, Zinta et al. highlight that the efficiency of N uptake and utilization depends on plant root development. Therefore, knowledge of genes involved in N metabolism is essential for plant breeding studies to increase plant tolerance to abiotic stress. Several studies are available on the response to nitrogen deficiency stress, but responses to N excess, common in agricultural systems with fertilizers, are limited. Smoczynska et al. had shown that the HvMADS transcription factor is down-regulated under N excess in barley roots, resulting in increased *HvBG1* expression and ABA release of ABA-glucose conjugates and consequent inhibition of root growth. ABA-induced tobacco roots, the expression of *NtPME029* was high (Sun et al.), which has ABA response elements (ABRE) and auxin-responsive region (AuxRR-core) in the promoter regions, indicating the involvement of this gene in the ABA and auxin pathways in mediating root development.

ABA is also involved in the ripening process of non-climacteric fruits such as blueberries. In the work of Wang et al., SUCROSE NON-FERMENTING1-RELATED PROTEIN KINASE-2 (SnRK2), an important component of the ABA signaling pathway, was shown to interact interacts with a positive anthocyanin regulator VcMYB1 and promotes the synthesis of this pigment in blueberries. Heterologous expression of *VcSnRK2.3* in *Arabidopsis* also induced anthocyanin pigmentation in seeds and seedlings.

Finally, post-transcriptional events such as ubiquitination and SUMOylation were shown to play important roles in regulating gene transcription, the quality of DNA replication and repair, and the abundance of short-lived regulatory proteins that are important for tolerance to various abiotic stresses and have been studied in detail by Singh et al.. The authors described the functions of ubiquitination and SUMOylation under drought stress, salinity, extreme temperatures, UV radiation, nutrient deprivation, ABA-mediated, flooding, and epigenetic regulation of stress responses and elucidated the molecular and enzymatic mechanisms of covalent protein modifications in plant immunity to abiotic stress.

## Author contributions

All authors listed have made a substantial, direct, and intellectual contribution to the work and approved it for publication.

